# Toxin-Based Therapeutic Approaches

**DOI:** 10.3390/toxins2112519

**Published:** 2010-10-28

**Authors:** Assaf Shapira, Itai Benhar

**Affiliations:** Department of Molecular Microbiology and Biotechnology, The George S. Wise Faculty of Life Sciences, Tel Aviv University, Ramat Aviv 69978, Israel; Email: assafsha@yahoo.com

**Keywords:** toxins, targeting, *pseudomonas* exotoxin A, diphtheria toxin, ricin, anthrax, immunotoxins, suicide gene

## Abstract

Protein toxins confer a defense against predation/grazing or a superior pathogenic competence upon the producing organism. Such toxins have been perfected through evolution in poisonous animals/plants and pathogenic bacteria. Over the past five decades, a lot of effort has been invested in studying their mechanism of action, the way they contribute to pathogenicity and in the development of antidotes that neutralize their action. In parallel, many research groups turned to explore the pharmaceutical potential of such toxins when they are used to efficiently impair essential cellular processes and/or damage the integrity of their target cells. The following review summarizes major advances in the field of toxin based therapeutics and offers a comprehensive description of the mode of action of each applied toxin.

## 1. Introduction

**Figure 1:**
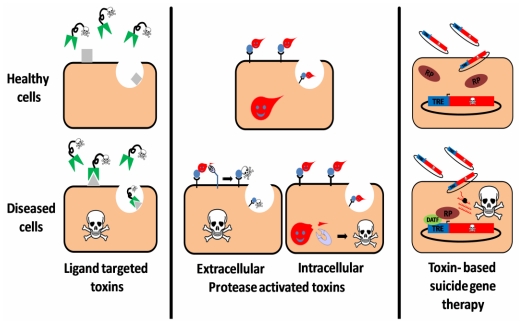
Three targeting strategies in toxin based therapy. Ligand targeted toxins: a ligand (antibody, antibody derivative, cytokine, *etc.*), which specifically binds to a disease related cell-surface antigen/receptor is linked to a toxic moiety, preferentially as a replacement to the natural cell binding domain of that toxin. Upon administration to patients, the construct selectively binds, is internalized and intoxicates diseased cells, sparing healthy cells that do not display the target on their surface. Protease activated toxins: the toxin is engineered to be cleaved and activated by a disease-related intracellular or extracellular protease. Toxin cleavage may enhance cell-binding and/or translocation, stabilization or catalytic activity of the toxic moiety specifically in protease expressing cells, leading to their eradication. Toxin based suicide gene therapy: a DNA construct, encoding for a toxic polypeptide whose expression is regulated by a specific transcription regulation element (TRE), is delivered to a heterogeneous cell population. However, intoxication occurs only in diseased cells that express an active disease-associated transcription factor (DATF) that specifically binds to the TRE and activates the transcription machinery (RP: RNA polymerase).

The secretion of polypeptides by prokaryotic and eukaryotic cells is an elaborate mechanism enabling the execution of essential processes like active modulation of the environment, enzymatic processing of nutrients and communication with other cells. However, a unique group of secreted polypeptides, the secreted toxins, plays a different role in maintaining the fitness of the organism, and have been perfected through evolution with the aim of damaging other living organisms. As such, toxins provide their producer with advantages such as enhanced defense capabilities or pathogenic competence. Most natural protein toxins can be divided into three major groups: 1. Toxins that damage the cell by disrupting membrane integrity; 2. Toxins that disrupt the normal electrical activity of the nervous system of the intoxicated organism; 3. Toxins that disrupt or interfere with cellular processes by virtue of an enzymatic activity. Members of groups 1 and 2 may affect the target cells by enzymatic or non-enzymatic activities. Some members of the third group, on which this review is focused, are extremely toxic polypeptides that have the capability of self translocation into the cell cytoplasm where they execute their activity that, in most cases, leads to death of the intoxicated cell. Scientific advances in the last decades facilitated the processing and manipulation of biological substances; among which are toxic polypeptides and their encoding genes. By using different strategies for directing toxic moieties to diseased cells/tissues (Figure 1), scientists have established a new niche in clinical research, called “toxin-based therapy”. 

The following review provides highlights of several prominent studies in this field; products from some of these studies have been evaluated clinically, or are currently undergoing clinical evaluation, mostly in context of oncological diseases. Constructs were classified by the targeting strategy used, namely surface antigen/receptor specific targeting (immunotoxins), transcriptional targeting (suicide genes) and protease specific targeting (Protease activated toxins). Emphasis has been put on constructs in which the toxic moiety is a derivative of the bacterial toxins produced by *Corynebacterium diphtheria* (Diphtheria toxin), *Pseudomonas aeruginosa* (Pseudomonas exotoxin A) and *Bacillus anthrachis* (Anthrax toxin); or plant produced toxins (ribosome inactivating proteins). For recent reviews about the botulinum toxin, a bacterial neurotoxin which is commonly applied in today’s medicine and is not covered in the following pages, see [1,2,3,4].

## 2. Ligand Targeted Toxins—Immunotoxins

The term “immunotoxin” classically refers to molecules which consist of a protein toxin linked to a targeting moiety derived from the immune system (such as an antibody or an antibody fragment); but frequently expanded to include other target-specifying ligands (such as a cytokine). The idea of development of a “magic bullet” that has a specific attraction to a disease-causing target, avoiding healthy body cells, was originally suggested by Paul Ehrlich over 100 years ago [5,6,7]. However, it was only in the 1970s that therapeutic agents composed of toxins conjugated to antibodies against cell surface antigens were shown to kill tumor cells [8,9]. Since then, many hybrid molecules consisting of a toxin coupled with a specific targeting antibody/ligand were developed; most of them are targeted against tumor cells [10] (Figure 1). 

**Figure 2:**
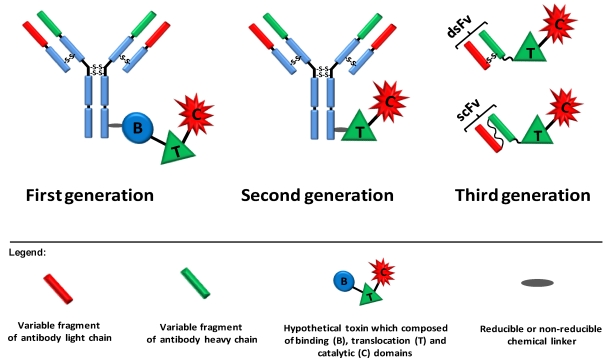
Three generations of immunotoxins. First generation immunotoxins were prepared by chemically conjugating antibodies/ligands to intact toxin units or to toxins with attenuated cell binding capability. Reducible or non-reducible chemical bonds/linkers were used for that purpose; the first was generally applied when the conjugation site was positioned on part of the toxin that translocates to the cytosol. In second generation immunotoxins, truncated toxins that lack a cell binding domain were chemically conjugated to a targeting moiety. In third generation immunotoxins, mostly produced in the bacterium *Escherichia coli*, the cell binding domain of the toxin is genetically replaced with a ligand or with the Fv portion of an antibody in which the light and heavy chain variable fragments are either genetically linked (scFv) or held together by a disulfide bond (dsFv).

First generation immunotoxins were prepared by chemically conjugating antibodies to natural‑intact toxin units or to toxins with attenuated cell binding capability. However, these constructs were heterogeneous and unspecific because of the multiplicity of potential sites available for chemical conjugation and as the presence of the cell binding domain of the toxin led to intoxication of “normal” cells, respectively. Immunotoxins of the second generation were also based on chemical conjugation between the targeting moiety and the toxin. Nevertheless, cumulative knowledge on the structure and function of the toxins enabled the removal of their native non-specific cell binding domain, generating much more target-specific immunotoxins when conjugated to monoclonal antibodies. Although more specific, and thus better tolerated by animals, immunotoxins from the second generation were still chemically heterogeneous and their large size hindered them from penetrating solid tumors. In order to avoid heterogeneity, improve tumor penetration and reduce production complexity and costs, recombinant DNA techniques were applied in the production of third generation immunotoxins. In these constructs, which are mostly produced in the bacterium *Escherichia coli*, the cell binding domain of the toxin is genetically replaced with a ligand or with the Fv portion of an antibody in which its light and heavy chain variable fragments are either genetically linked (scFv) or held together by a disulfide bond (dsFv) (Figure 2). 

Among the bacterial toxins that were used for the construction of immunotoxins, the most common are diphtheria toxin and *pseudomonas* exotoxin A, which are naturally produced by the Gram-positive, aerobic *Corynebacterium diphtheria* and by the Gram-negative, aerobic *Pseudomonas aeruginosa*, respectively. Plant derived toxins, which belong to a group of toxins denoted “ribosome inactivating proteins” (RIPs), were also used in the preparation of immunotoxins. Among them, ricin, saporin and pokeweed antiviral protein (PAP), produced by *Ricinus communis*, *Saponaria officinalis* and *Phytolacca americana*, respectively, were most commonly used. All of these toxins are extremely potent and exert their toxicity by enzymatically inhibiting the protein synthesis machinery of eukaryotic cells (for additional reviews on immunotoxins, see [10,11,12,13,14,15,16]).

In the next chapter, selected immunotoxins will be classified by their toxic moiety (diphtheria toxin, *pseudomonas* exotoxin A or RIPs derivatives), and a short review on the mechanism of action of their coupled toxins will be followed by a brief description of their disease-causing target, targeting antibody/ligand, and current status of clinical trials. Information about these and other clinically evaluated immunotoxins is summarized in Table 1. For ligand targeted toxins, we provide detailed examples of toxin based therapies that were evaluated in patients and do not detail the hundreds of examples of molecules that were evaluated pre-clinically.

**Table 1:** Clinically evaluated/under evaluation immunotoxins.

Construct Name	Targeting Moiety	Toxic Moiety	Toxin Source	Target	Indication	Clinical Trial Phase	References
**DAB_389_IL2 (Denileukin Diftitox)**	IL˒	DAB_389_	DT	IL˒R	CTCL, NHL, CLL, NSCLC, GVHD, psoriasis, melanoma, ovarian, breast, kidney cancers	I,II,III,IV *FDA approvedFor CTCLtreatment	[17,18,19,20,21,22,23,24,25]
**DAB_486_IL2**	IL-2	DAB_486_	DT	IL-2R	NHL, HD, CLL, CTCL, KS, RA	I/II	[26,27,28,29,30,31]
**Tf-CRM107 (TransMID)**	Transferrin	CRM107	DT	TfR	Brain and CNS tumors	I, III	[32,33,34]
**DT388-GM-CSF**	GM-CSF	DT_388_	DT	GM-CSFR	AML	I	[35]
**DAB_389_EGF**	EGF	DAB_389_	DT	EGFR	EGFR-expressing carcinoma	I/II	[11]
**A-dmDT390-bisFV (UCHT1)**	bisFv	DT_390_	DT	CD3ε	T-cell lymphoma/leukemia	I/II	[36,37]
**DT388-IL3**	VariantIL-3	DT_388_	DT	IL-3R	AML, MDS	I/II	[38]
**OVB3-PE**	MAb	Full length PE	PE	Ovarian antigen	Ovarian cancer	I	[39]
**ERB-38**	dsFv	PE38	PE	erbB2/ HER2	Breast, esophageal cancers	I	[40]
**SS1(dsFv)PE38 (SS1P)**	dsFv	PE38	PE	Mesothelin	Mesothelioma, ovarian, pancreatic cancers	I	[41,42]
**B3(Fv)-PE38 (LMB-7)**	scFv	PE38	PE	Lewis Y	Adenocarcinoma	I	[10]
**LMB-1**	MAb	PE38	PE	Lewis Y	Adenocarcinoma	I	[43]
**RFB4(dsFv)-PE38 (BL22/CAT3888)**	dsFv	PE38	PE	CD22	NHL, CLL, HCL, ALL	I,II	[44,45,46,47]
**LMB-2**	scFv	PE38	PE	CD25	Leukemia, lymphoma	II	[48]
**scFv(FRP5)-ETA**	scFv	PE40	PE	erbB2 /HER2	Melanoma, Breast, colon cancers	I	[49,50]
**TP40**	TGFα	Modified PE40	PE	EGFR	Bladder cancer	I	[51]
**TP38**	TGFα	PE38	PE	EGFR	Glioblastoma	II	[52,53,54]
**BR96sFv-PE40 (SGN-10)**	scFv	PE40	PE	Lewis Y	Adenocarcinoma	I	[55]
**B3(dsFv)-PE38 (LMB-9)**	dsFv	PE38	PE	Lewis Y	Adenocarcinoma	I	[10]
**IL4(38-37) PE38KDEL (NBI-3001)**	Circularly permuted IL-4	Modified PE38	PE	IL-4R	Brain, CNS, kidney, lung, breast cancers	I,II	[56,57,58]
**Mutated RFB4(dsFv)-PE38 (HA22/CAT-8015)**	dsFv	PE38	PE	CD22	HCL, ALL, NHL CLL, PLL, SLL	I	[59,60]
**IL13-PE38QQR (cinterdekin besudotox)**	IL-13	Modified PE38	PE	IL-13R	Glioma	I/II, III	[61,62,63,64]
**RFB4-Fab'-dgA**	Fab'	Deglycosylated RTA	Ricin	CD22	B-NHL	I	[65]
**RFB4-dgA (IMTOX-22)**	MAb	Deglycosylated RTA	Ricin	CD22	B-NHL, CLL	I	[66,67]
**HD37-dgA (IMTOX-19)**	MAb	Deglycosylated RTA	Ricin	CD19	NHL	I	[68,69]
**RFB4-dgA + HD37-dgA (Combotox)**	MAb	Deglycosylated RTA	Ricin	CD22, CD19	NHL, ALL	I	[70,71]
**RFT5-dgA (IMTOX-25)**	MAb	Deglycosylated RTA	Ricin	CD25	HD, CTCL, melanoma, GVHD	I,II	[72,73,74,75,76]
**Ki-4.dgA**	MAb	Deglycosylated RTA	Ricin	CD30	HD, NHL	I	[75,77]
**Anti-B4-bR**	MAb	Blocked ricin	Ricin	CD19	B-NHL	II	[78,79,80,81]
**Anti-CEA-bR**	MAb	Blocked ricin	Ricin	CEA	Colorectal cancer	I/II	[82]
**N901-bR**	MAb	Blocked ricin	Ricin	CD56	SCLC	I	[83,84,85]
**Anti-CD7-dgA (DA7)**	MAb	Deglycosylated RTA	Ricin	CD7	T-NHL	I	[86]
**Anti-CD3-dgA +Anti-CD7-dgA**	MAb	Deglycosylated RTA	Ricin	CD3, CD7	GVHD	I/II	[87]
**CD5-IC, CD5 Plus**	MAb	RTA	Ricin	CD5	RA, SLE, diabetes mellitus	I,II	[88,89,90,91,92]
**H65-RTA**	MAb	RTA	Ricin	CD5	CTCL, GVHD	I, I/II	[93,94,95]
**T101-RTA**	MAb	RTA	Ricin	CD5	CLL	I	[96,97,98]
**MDX-RA**	MAb	RTA	Ricin	Human lens epithelial antigen	Posterior capsule opacification (secondary cataract)	III	[99,100,101]
**XomaZyme-Mel(XMMME-001-RTA)**	MAb	RTA	Ricin	Melanoma antigen	Melanoma	I/II	[102,103,104,105,106,107]
**XomaZyme-791(79IT/36-RTA)**	MAb	RTA	Ricin	72kDa TAA	Colorectal cancer	I	[108,109,110]
**454A12-rRA**	MAb	RTA	Ricin	TfR	Leptomeningeal neoplasia	I	[111]
**260F9-rRTA**	MAb	RTA	Ricin	55 kDa breast cancer antigen	Breast cancer	I	[112,113]
**B43-PAP**	MAb	PAP	PAP	CD19	ALL	I	[114]
**TXU-PAP**	MAb	PAP	PAP	CD7	HIV-1 infection	I	[115]
**BER-H2-Sap6**	MAb	Saporin	Saporin	CD30	HD	I	[116]
**HUM-195/rGel**	MAb	Gelonin	Gelonin	CD33	AML, CML	I	[117]
**BDI-1-MD**	MAb	Momordin	Momordin	Bladder carcinoma antigen	Bladder cancer	I	[118]

Abbreviations: MAb: monoclonal antibody; dsFv: disulfide-stabilized Fv antibody fragment; scFv: A single-chain (genetically linked) variable fragment; bisFv: two Fv fragments connected via a disulfide bond; Fab': fragment antigen-binding (one constant / one variable domain of each heavy and light chain connected by a disulfide bond); IL(R): interleukin (receptor); DT: diphtheria toxin; DAB_389_, DAB_486_, DT_388_, DT_390_: truncated forms of DT that lack receptor-binding activity; CRM107: a mutated full-length diphtheria toxin that lack receptor-binding activity; PE: *pseudomonas* exotoxin A; PE38, PE40: truncated forms of PE that lack the receptor-binding domain Ia; RTA: ricin toxin A; HIV: human immunodeficiency virus; CTCL: cutaneous T cell lymphoma; NHL: non-Hodgkin’s lymphoma; MDS: myelodysplastic syndrome; ALL: acute lymphoblastic leukemia; SLL: small lymphocytic lymphoma; GVHD: graft *versus* host disease; CNS: central nervous system; EGF(R): epidermal growth factor (receptor); TGF(R): transforming growth factor (receptor) AML: acute myelogenous leukemia; CML: chronic myelogenous leukemia; CLL: chronic lymphocytic leukemia; HD: Hodgkin’s disease; HCL: hairy cell leukemia; PLL: prolymphocytic leukemia; (N)SCLC: (non) small cell lung cancer; TAA: tumor associated antigen; TfR: transferrin receptor; CSF: cerebrospinal fluid, PAP: pokeweed antiviral protein; RA: rheumatoid arthritis; SLE: systemic lupus erythematosus.

### 2.1. Diphtheria Toxin Based Immunotoxins

#### 2.1.1. Diphtheria Toxin—Mechanism of Action

**Figure 3:**
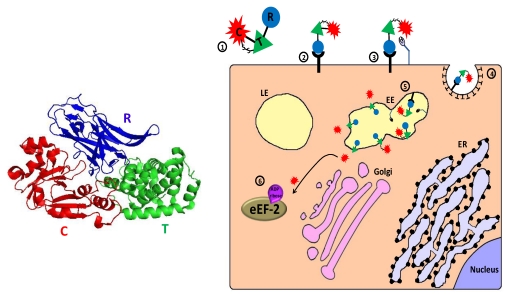
Main entry route and mechanism of action of diphtheria toxin. 1. The toxin is secreted as one polypeptide which is composed of three functional domains: the N terminal catalytic domain ((C), also referred to as DTA/DT-A), the translocation domain (T) and the receptor binding domain (R) (see 3D structure (PDB Entry: 1f0l). In the left panel, the colors of the subunits correspond to those in the scheme). In addition, a disulfide bond bridges the C and T domains; 2. The toxin binds via its R domain to a cellular receptor (heparin binding epidermal growth factor precursor); 3. Cell-surface furin protease cleaves the polypeptide chain between the C and T domains that remain linked by a disulfide bond; 4. The toxin-receptor complex is internalized into clathrin coated pits; 5. In the lumen of the early endosome (EE), furin protease cleaves toxin molecules that escaped cell-surface cleavage. The T domain undergoes acidic-induced conformational change, inserted into the endosome membrane and forms a channel through which the catalytic domain can translocate into the cytoplasm where reduction of the interdomain bridging disulfide bond occurs; 6. In the cytoplasm, the catalytic domain inactivates eukaryotic translation elongation factor 2 (eEF2) by ADP-ribosylation, which causes translation inhibition and consequently cell death.

Diphtheria toxin (DT), secreted by pathogenic strains of the bacterium *Corynebacterium diphtheria*, is the prototype for the family of ADP-ribosylating toxins. It belongs to a group of toxins called AB toxins that consist of two fragments (A and B). The B fragment is responsible for cell entry (binding to a cell surface receptor and subsequent translocation into the cell cytoplasm) while the internalized A fragment intoxicates the cell by virtue of its enzymatic activity [119,120]. The toxin is secreted as a single protein of 535 amino acids and is composed of three functional domains: the N terminal domain (residues 1–193) represents the catalytic (C) A fragment/domain (DTA/DT-A). The C terminal portion of the toxin (amino acids 194–535) represents the B fragment and is divided into two functional domains: the translocation domain (T) (amino acids 202–378) and the receptor binding domain (R) (amino acids 386–535) [121]. The native diphtheria toxin binds, via its R domain, to heparin binding epidermal growth factor precursor on the cell membrane, where it is cleaved by cell-surface furin or furin-like protease [122,123]. The di-chain protein that is still linked by a single disulﬁde bond between cysteine 186 and cysteine 201 is internalized into clathrin coated pits and reaches the lumen of a developing endosome (where furin-mediated cleavage of toxin molecules that escaped cleavage by cell-surface proteases, may occur [122]). Upon endosome acidification, the T domain undergoes a conformational change that leads to exposure of hydrophobic areas that are inserted into the membrane, forming a channel through which the catalytic domain translocates and escapes from the endosome, probably with the aid of cytosolic factors [124,125,126,127,128,129]. In the cell cytoplasm, the catalytic domain exerts its toxic activity by transferring adenosine di-phosphate-ribose (ADP-ribose) moiety from nicotinamide dinucleotide (NAD) to a modified histidine residue (diphthamide) at position 715 in the eukaryotic translation elongation factor (eEF2). This action results in the inactivation of the latter, inhibition of protein synthesis, and programmed cell death [13,130,131,132,133] (Figure 3). It was also reported that delivery of a single molecule of the catalytic domain into the cytosol is sufficient to kill a cell, demonstrating the extreme potency of this bacterial toxin [134]. 

#### 2.1.2. Targeting IL-2 Receptor: Denileukin Diftitox (Ontak)

Denileukin Diftitox, which is also named “Ontak” or “DAB389 IL-2”, is a fusion protein designed to direct a truncated form of diphtheria toxin to cells that express the high-affinity IL-2 receptor, (consisting of the following subunits: CD25 (IL-2Rα), CD122 (IL-2Rβ), and CD132 (IL-2Rγ)), which is present in many different hematologic malignancies like adult T cell leukemia (ATL), chronic lymphocytic leukemia, Hodgkin’s and non-Hodgkin’s lymphomas, cutaneous T cell lymphoma (CTCL) and other leukemias and lymphomas [10,135,136,137,138,139]. The immunotoxin is comprised of a genetic fusion between a truncated form of DT (first 388 amino acids, “DAB_389_”), in which the natural receptor binding domain of the toxin was replaced by the cytokine interleukin-2 (IL-2) [140]. Phase I testing was conducted on patients with T-cell lymphoma (CTCL) (n = 35), other non-Hodgkin's lymphomas (NHL) (= 17), or Hodgkin's disease (HD) (n = 21). The drug, which was administrated by intravenous infusion, produced five complete (CR) and eight partial (PR) remissions in patients with CTCL with one CR and two PR occurring in NHL. No response was documented in patients with HD. The dose-limiting toxicity in these trials was asthenia [141]. In the pivotal phase III trial, 30% of 71 patients with CTCL treated with Denileukin Diftitox had an objective response (20% partial response; 10% complete response) [18]. since the FDA approval of ONTAK as the first immunotoxin for treatment of advanced CTCL in 1999, the drug was tested for treatment of other malignant and non-malignant diseases like B-cell NHL [23], B-cell chronic lymphocytic leukemia (CCL) [19] panniculitic lymphoma [142], psoriasis [17,143] and Graft-versus-host disease (GVHD) [21]. Responses were observed in all of these trials.

#### 2.1.3. Targeting Granulocyte-Macrophage Colony Stimulating Factor Receptor: DT388-GM-CSF

The majority of acute myeloid leukemia (AML) blast cells express the granulocyte-macrophage colony-stimulating factor (GM-CSF) receptor [144]. In order to target these cancerous cells, the human GM-CSF was fused to DT388, a truncated DT toxin, replacing its natural receptor binding domain [145,146]. The resulting molecule, DT388-GM-CSF (DTGM), was tested on 31 patients who were resistant to chemotherapy. Among them, one had a complete remission and two had partial remissions following treatment with the drug that was administrated by intravenous (i.v.) infusion. Liver failure or transient hepatic encephalopathy were observed in two patients, possibly as a result of inflammatory cytokine release from liver Kupffer cells (DT388-GM-CSF does not directly bind or damage hepatocytes) [35]. 

#### 2.1.4. Targeting Transferrin Receptor: Tf-CRM107 (TransMID)

Transferrin receptors (TfRs) are overexpressed on rapidly dividing cells and various tumor cells. While relatively scarce in healthy brain tissue, intense expression of TfRs can be found on tumor cells of glioblastoma multiforme (GBM's) [147]. Tf-CRM107, which is also called “transMID” is a conjugate protein of a mutant diphtheria toxin that lacks receptor-binding activity (CRM107) [148], linked by a thioester bond to human transferrin (Tf) [149]. In Phase I clinical trials, TF-CRM107 was delivered by high-flow interstitial microinfusion into the tumor region and reduction in tumor volume occurred in nine of 15 patients who could be evaluated, including two complete responses. No symptomatic systemic toxicity occurred [32]. In the phase II clinical study, Tf-CRM107 treatment resulted in a 35% response rate: Of the 34 patients evaluable for efficacy, there were a total of five complete responders and seven partial responders. Infusions of Tf-CRM107 resulted in symptomatic progressive cerebral edema in eight of the total enrolled 44 patients (14%) that were responsive to medical management. Seizures were seen in three patients who responded to anticonvulsant therapy [33]. However, a conditional power analysis in phase III determined that Tf‑CRM107 was unlikely to improve overall patient survival compared with the current standard of care, and it was decided to terminate the trial and further clinical development of the drug [34].

### 2.2. Pseudomonas Exotoxin A Based Immunotoxins

#### 2.2.1. *Pseudomonas* Exotoxin A—Mechanism of Action

**Figure 4:**
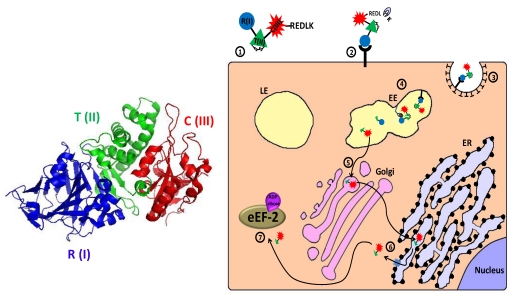
Main entry route and mechanism of action of *Pseudomonas* exotoxin A. 1. The secreted *pseudomonas* exotoxin A (PE) toxin can be divided into three main structural and functional domains: the N terminal receptor (R) binding domain I, translocation (T) domain II and the catalytic (C) domain III (see 3D structure (PDB Entry: 1ikq). In the left panel, the colors of the subunits correspond to those in the scheme. For the sake of simplicity, translocation domain II was extended to contain subdomain Ib). A single disulfide bond bridges between cysteines 265 and 287 within domain II; 2. Following removal of a C terminal lysine residue by plasma carboxipeptidase, the toxin binds to its cell-surface receptor (CD91, also called α2MR/LRP); 3. The toxin is internalized mainly via clathrin-coated pits; 4. In the early endosome (EE), the toxin undergoes conformational change and is cleaved by the protease furin in a furin sensitive loop, in domain II. The two cleavage products remain linked by the intradomain disulfide bond; 5. Following reduction of the disulfide bond, the enzymatically active C terminal fragment, which is composed of domain III and about two thirds of domain II, is routed to the trans-Golgi network where it binds via its C terminally exposed REDL sequence to KDEL receptor and travels to the endoplasmic reticulum (ER); 6. In the ER, sequences in domain II mediate the retro‑translocation of the polypeptide via the Sec61p translocon into the cytoplasm; 7. The catalytic domain inactivates eukaryotic translation elongation factor 2 (eEF2) by ADP‑ribosylation, which causes translation inhibition and consequently cell death.

*Pseudomonas* exotoxin A (abbreviated as PE or ETA) is a 613 amino acid polypeptide secreted by the bacterium *Pseudomonas aeruginosa* as one of its virulence factors [150]. Like diphtheria toxin, it belongs to the family of ADP-ribosylating toxins [151] and to the group of AB toxins (see description of diphtheria toxin above). The toxin can be divided into three main structural and functional domains: The N-terminal domain Ia (aa 1–252) is responsible for cell recognition. Domain II (aa 253–364) is required for the translocation of the toxin across cellular membranes. The exact function of the structural domain Ib (aa 365–404) is not fully understood. The last four residues (aa 400–404) of domain Ib together with domain III (aa 405–613) form the catalytic subunit of the protein [152,153]. After the C-terminal Lysine 613 is removed by a plasma carboxypeptidase [154], leaving the terminus REDL, the toxin binds via its cell-binding domain Ia to CD91, also called alpha2‑macroglobulin receptor/low-density lipoprotein receptor-related protein (*α*2MR/LRP), on the surface of the cell [155]. The toxin is then internalized and enters early endosomes mainly via clathrin-coated pits, but also via caveosomes, following association with detergent-resistant microdomains [155,156]. In the acidic environment of the endosome, PE dissociates from its receptor, undergoes a conformational change, and is cleaved by the cellular protease furin in a furin-sensitive loop in domain II of the toxin [123,157,158]. Following reduction of the single disulfide bond which holds the proteolytic fragments together [159], the enzymatic active C’ 37 kDa fragment travel in a Rab9-dependent route to the trans-Golgi network (TGN) [160]. There, its C terminal exposed KDEL‑like sequence (REDL) binds the KDEL intracellular sorting receptor and the fragment travels to the endoplasmic reticulum (ER) [161,162,163]. Alternatively, lipid sorting to the ER may occur [156]. In the ER, sequences in translocation domain II mediates the translocation of the 37 kDa fragment to the cytoplasm in a process that probably involves the subversion of the ER-associated degradation (ERAD) pathway and a retrograde transport via the Sec61p translocon. Escaping, at least in part, from proteosomal degradation may be attributed to the low lysine content of the enzymatically active C terminal 37 kDa fragment [164,165,166,167,168,169]. In the cytosol, the APD-ribosylation enzymatic activity of domain III inactivates eEF-2 in a similar way to that of diphtheria toxin (see above), leading to protein synthesis inhibition and programmed cell death [130,132,170,171,172,173] (Figure 4). 

#### 2.2.2. Targeting the CD25 Subunit of IL2-Receptor: LMB-2

For the construction of PE-based immunotoxins, the receptor binding domain Ia can be replaced by an antibody, antibody derivative, or a ligand, which preferentially binds to tumor-associated antigen/receptor. The resulting truncated form of PE is designated PE40, indicating its molecular weight (40 kDa). In addition, a large part of domain Ib can be deleted without effecting cytotoxicity, generating a smaller form of the modified toxin, which is denoted PE38 (38 kDa). 

In order to target CD25 expressing cells (low-affinity IL2 receptor, see above) regardless of the presence of other IL2R subunits (CD25 is generally expressed to a much greater extent relative to CD122 and CD132 on most malignant cell types [137,138]), PE38 was fused to a single-chain form of the anti-CD25 monoclonal antibody anti-Tac [174,175,176]. The resulting immunotoxin, Anti–TacFv–PE38 (LMB-2), demonstrated promising results in pre-clinical trials toward CD25^+^ cells and tumor xenografts in nude mice [177,178,179]. In a Phase I trial, LMB-2 was administrated intravenously to 35 patients with chemotherapy-resistant leukemia, lymphoma and HD, resulting in 1/35 (3%) complete and 7/35 (20%) partial remissions. The most common toxicities included transaminase elevations that were associated with fever, possibly as an outcome of cytokine release. Six of the 35 patients made neutralizing antibodies after one cycle, preventing them from receiving re‑treatment [180,181,182]. In Phase II trials on patients with metastatic melanoma, administration of LMB-2 has led to a transient partial reduction in circulating and tumor-infiltrating CD25^+^ T–regulatory (Treg) cells which are able to suppress the ability to vaccinate against self/tumor antigens [183]. However, LMB-2 therapy did not augment the immune response to peptide based cancer vaccines [184]. Other Phase II trials are currently underway in patients with CD25^+^ Chronic Lymphocytic Leukemia (CLL), Cutaneous T cell lymphoma (CTCL) and Hairy Cell Leukemia (HCL).

#### 2.2.3. Targeting CD22: BL22

CD22 is a 135-kDa phosphoglycoprotein adhesion molecule present on the surface of B cells, including human B-cell lymphomas and leukemias [185,186,187,188,189]. RFB4(dsFv)- PE38 (BL22) is a stable immunotoxin targeted against CD22 expressing cells, and is composed of disulfide stabilized Fv regions (dsFv) of the anti-CD22 monoclonal antibody RFB4 [190] fused to PE38 [191]. Clinical trials with *i.v.* administrated BL22 in adults with hairy cell leukemia resistant to purine analogue therapy, produced promising results with 19 complete remissions (61%) and six partial responses (19%) in 31 patients in Phase I [45], and 25% complete remission, 25% partial response after one cycle of treatment in Phase II (n = 36) [46]. The most common toxicities included hypoalbuminemia, transaminase elevations, fatigue, edema and reversible grade 3 hemolytic uremic syndrome, not requiring plasmapheresis. A recent Phase I clinical trial that was conducted for pediatric subjects with CD22^+^ ALL and non–Hodgkin lymphoma [47] showed that the treatment was associated with an acceptable safety profile and adverse events were rapidly reversible. No maximum tolerated dose was defined, and although no responses were observed, transient clinical activity was seen in most subjects [192,193,194]. 

#### 2.2.4. Targeting the Le^Y^ Antigen: LMB-1

Lewis Y (Le^Y^), a type 2 blood group related oncofetal carbohydrate antigen is expressed on nearly 70% of human epithelial carcinomas [195,196,197]. The LMB-1 immunotoxin consists of the anti–Lewis Y monoclonal antibody B3 [198] conjugated to PE38. In a Phase I clinical trial, the immunotoxin was tested on 38 patients with Le^Y^ expressing carcinomas of breast, ovarian, colon, esophagus, stomach and ampulla of Vater. A complete remission was observed in a patient with metastatic breast cancer and a greater than 75% tumor reduction was observed in a colon cancer patient following systemic administration of the immunotoxin. The major toxicity was vascular leak syndrome ascribed to endothelial damage, probably due to binding of the B3 antibody to Le^Y^ antigen which is present in small amounts on endothelial cells [199]. Later developments of Lewis Y targeted toxins produced the recombinant immunotoxins B3(Fv)-PE38 (LMB-7), B3(dsFv)-PE38 (LMB-9) and BR96-Fv-PE40 (SGN-10) which were clinically evaluated in patients with Lewis Y-expressing malignancies. However, no significant antitumor activity was observed in these trials [10,200]. 

### 2.3. Ribosome Inactivating Proteins Based Immunotoxins

#### 2.3.1. Ribosome Inactivating Proteins—Mechanism of Action

Ribosome inactivating proteins (RIPs) are a group of glycosylated and non-glycosylated enzymes with *N*-glycosidase activity that were initially detected in higher plants, but have also been found in fungi, algae and bacteria (for comprehensive reviews, see [201,202,203,204,205,206,207]). RIPs may be present in one or more tissues of the plant, and their expression is enhanced in senescence and under various stress conditions [208,209,210,211,212,213,214] including microorganisms and viral infections [215,216,217]. RIPs are artificially divided into three groups on the basis of their structure and mode of activation: type I RIPs are single chain basic proteins of about 30 kDa with enzymatic activity. Some well known members of this group are saporin (from *Saponaria officinalis*), pokeweed antiviral protein (PAP) (*Phytolacca americana*) and gelonin (*Gelonium multiforum*). Type II RIPs, like ricin (from *Ricinus communis*) and abrin (*Abrus precatorius*), are heterodimeric proteins consisting of an enzymatically active A chain of about 30 kDa linked through a disulfide bond to a B chain of approximately 35 kDa which has the properties of a lectin. Type III RIPs, like the maize and the barley proteins b-32 and JIP60, respectively, are synthesized as inactive precursors (proRIPs), which lacks a lectin moiety and are activated by proteolytic processing which includes the removal of terminal sequences and a short inhibitory internal peptide [201,206,207,208,218,219,220].

**Figure 5:**
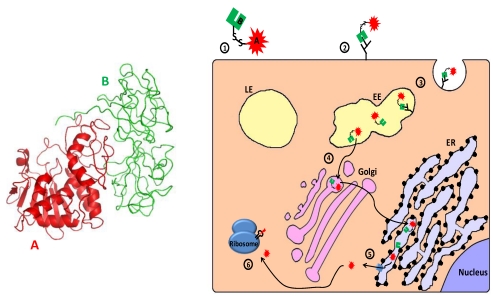
Main entry route and mechanism of action of ricin. 1. Ricin toxin is translated as a single glycosylated polypeptide that is composed of a catalytic A domain and a lectin B domain (see 3D structure (PDB Entry: 2aai) in the left panel; the colors of the subunits correspond to those in the scheme). In the producing plant, a small peptide that links the A and B domains is removed, and the A and B chains remain associated via a single disulfide bond; 2. The toxin binds through the lectin B chain to cell-surface galactose or N‑acetylgalactosamine residues on glycoproteins and glycolipids; 3. Cell-surface bound ricin is internalized by clathrin-dependent as well as clathrin-independent endocytosis and reaches the early endosome (EE); 4. The toxin travels backward through the Golgi to the endoplasmic reticulum (ER), where its’ disulfide linked chains are separated; 5. The catalytic A chain (RTA) is retro-translocated via the Sec61p translocon into the cytoplasm; 6. The catalytically active RTA irreversibly damages ribosome by removing a specific adenine from a conserved 28S rRNA loop (“sarcin/ricin loop”–SRL), which causes translation inhibition and consequently cell death.

Ricin, the prototype of type II RIPs, is a glycosylated heterodimer that binds through its lectin B‑chain to galactose or N-acetylgalactosamine residues on glycoproteins and glycolipids which are present on the surface of most eukaryotic cells [221,222,223,224,225,226]. In addition, certain cells like macrophages and rat liver endothelial cells that express surface mannose receptors were demonstrated to bind ricin also through its own oligosaccharide side chains [227,228,229,230,231,232]. The cell surface-bound ricin is internalized by clathrin-dependent as well as clathrin-independent endocytosis and travels backward from the Golgi to the ER, where its disulfide linked chains are separated by protein disulfide isomerase. As in the case of PE, ricin is thought to subvert the ERAD pathway, exploiting it for the retrograde transport of the enzymatically active A chain (RTA) into the cell cytosol through the Sec61p translocon. Because of its paucity of lysines, RTA may escape, at least in part, degradation by the proteosome [167,169,233,234,235,236,237,238,239,240,241,242] (Figure 5). Type I and type III RIPs lack the cell-binding lectin B chain and are thus generally much less toxic than type II RIPs. However, like ricin, some glycosylated type I RIPs may bind to carbohydrate receptors on the cell surface, and binding of type I RIPs to low density lipoprotein (LDL) receptor related protein (*α*2MR/LRP), (which also binds PE) has also been demonstrated [243,244,245]. In addition, coupling type I RIPs (which are equivalent to the enzymatically active A chain of type II RIPs) to a carrier that is capable of binding cells renders the conjugate highly cytotoxic [206,246,247,248,249]. The specific mechanism by which type I RIPs gain entry into the cell cytosol remains unclear, but is probably different from that of type II RIPs like ricin: Experiments with the type I RIP, saporin, indicated that it does not rely on Golgi-mediated retrograde transport and ERAD and may involve toxin translocation to the cytoplasm from the endosomes [242,250]. 

Once in the cytosol, the enzymatic moiety of RIPs irreversibly damages ribosomes by removing a specific adenine (corresponding to residue A4324 in rat 28S rRNA) from a GAGA sequence in a conserved 28S rRNA loop, the so called “sarcin/ricin loop” [251,252,253]. This modification renders the ribosome unable to interact with elongation factors 2 (eEF2), resulting in inhibition of translation and ultimately apoptotic cell death [201,254,255,256,257,258,259,260,261,262,263,264]. In addition to their classical ribosome-inactivating glycosidase activity, other activities and enzymatic properties were associated with RIPs: antiviral activity [265], depurination of (non-ribosomal) RNA and adenine DNA glycosylase activity [266,267,268,269,270], deoxyribonuclease activity [271,272,273,274,275,276,277,278], ribonuclease activity [279,280], removal of adenine from poly(ADP-ribosyl)ated poly(ADP-ribose) polymerase (an enzyme involved in DNA repair) [281,282], depurination of the capped RNA template [283,284], superoxide dismutase (SOD) activity [285,286,287] and phospholipase activities [288]. The involvement of these non-classical RIP activities in cytotoxicity is debatable, though accumulating evidence suggests that ribosome inactivation is not the sole means by which RIPs execute their toxic effects [264,289,290,291,292,293,294,295]. 

#### 2.3.2. Targeting CD25 and CD30: RFT5-dgA and ki-4.dgA.

CD25 and CD30 are lymphoid activation markers which are highly expressed on the surface of Hodgkin's lymphoma cells and only present on a minority of normal human cells [135,296,297,298]. In order to target these cells, two immunotoxins, RFT5-dgA (IMTOX25) and ki-4.dgA, were constructed. The toxic moiety in these immunotoxins was a chemically deglycosylated form of ricin A chain (dgA) (deglycosylation was demonstrated to minimize nonspecific carbohydrate-receptors mediated uptake of RTA based immunotoxins by reticuloendothelial cells in the liver [299,300,301,302,303,304,305]) linked to the targeting moieties RFT5 (anti-CD25) and ki-4 (anti-CD30) monoclonal antibodies, respectively. Phase I/II trials with *i.v.* administrated RFT5-dgA on 18 patients with refractory Hodgkin's disease (HD), resulted in two partial remissions (PR), one minor response (MR) and five stable diseases (SD) [75]. In a Phase I study of Ki-4-dgA on 15 patients, one PR, one MR and two SD were observed. Dose-limiting toxicities were related to vascular leak syndrome, consisting of edema, tachycardia, dyspnea, weakness and myalgia [75]. A Phase II study evaluating the side effects and efficiency of RFT5-dgA (IMTOX25) treatment in patients with relapsed or refractory cutaneous T-cell non-Hodgkin lymphoma (CTCL) was completed recently, and the immunotoxin is currently evaluated as a treatment for metastatic melanoma (http://clinicaltrials.gov/). 

#### 2.3.3. Targeting CD22 and CD19: RFB4-dgA and HD37-dgA

CD22 and CD19 are cell surface glycoproteins expressed on normal and malignant B cells [306,307,308,309]. For targeting B-cell lymphoma cells, the anti CD22 monoclonal antibody RFB4 [190,310] and the anti CD19 monoclonal antibody HD37 [311] were conjugated to dgA. In a Phase I clinical trial with the immunotoxin RFB4-dgA (IMTOX-22), five of 24 evaluable patients with B-cell lymphoma showed a partial response and one showed a complete response. Similar results were obtained when the drug was administrated as a continuous infusion instead of intermittent bolus [67,312]. Partial and complete responses were also obtained in a Phase I trial on patients with non-Hodgkin's B-cell lymphoma treated with the immunotoxin HD37-dgA (IMTOX-19) [69]. Vascular leak syndrome (VLS) was a common dose-related toxicity in these studies.

## 3. Toxin Based Suicide Gene Therapy

The delivery of genetic material into target cells for the purpose of gaining a therapeutic effect is generally known as “gene therapy” (for review, see [313,314,315,316,317,318,319,320,321,322,323]). Advances in molecular biology, virology and nanotechnology in the past decade enabled the development of a variety of viral and non‑viral gene delivery systems [324,325,326]. When the death of the target cell is the desired therapeutic outcome of the transgene delivery, the process is termed “suicide gene therapy” and involves delivery of genes whose products are either toxic, proapoptotic or have the ability to activate precursors of toxic drugs (“pordrugs”) [327,328,329,330,331]. In order to minimize damage to healthy tissue, a specific targeting mechanism must be applied and transcriptional targeting is a very common strategy. The method is based on positioning the suicide gene to be transferred under the transcriptional regulation of a promoter/element which is specifically or at least preferentially active in the target tissue [332,333,334,335,336,337,338]. If transcription of a toxin encoding gene is controlled by such a target specific promoter, eradication of undesirable cell population is feasible with minimal collateral damage (Figure 1). The next chapter reviews some of the prominent toxin-based suicide gene therapy studies and developments which are classified by their target disease; the majority of them were evaluated at a preclinical level and aimed at cancer therapy. Information about suicide gene constructs developed in these and other studies are summarized in Table 2.

**Table 2:** Preclinically and clinically evaluated/under evaluation toxin-based suicide genes.

Construct Name	Transcription Regulatory Element	Toxin	Delivery Vector	Disease	Clinical Trial Phase	Reference
**Ad5-PSE/PSA-DT-A**	PSA *	DTA	Adenovirus	Prostate cancer	Preclinical phase	[339]
**Ad-PSA/FLP +Ad-RSV/FRT2neo/DT-A**	PSA *	DTA	Adenovirus	Prostate cancer	Preclinical phase	[340]
**C32-PSA/DT-A**	PSA *	DTA	Cationic polymer	Prostate cancer	Preclinical phase	[341]
**pTHA-47, pTHA-49**	hCG (α or β subunits) *	DTA	Naked DNA-electroporation	Ovarian cancer	Preclinica lphase	[342]
**pHE-4/DT-A,117-MSLN/DT-A**	HE4, MSLN *	DTA	Cationic polymer	Ovarian cancer	Preclinica lphase	[343]
**DTA-H19 (BC-819)**	H19 *	DTA	Naked DNA, Cationic polymer	Ovarian, bladder, pancreatic cancers	I/II, II	[344,345,346]
**DTA-TER, DTA-TERT**	hTER, hTERT *	DTA	Naked DNA- CaPO4 precipitate	Bladder cancer	Preclinical phase	[347]
**HIV-DT-A**	HIV Tat and Rev cis-acting responsive sequences	DTA	Retrovirus, cationic liposomes	HIV-1 infection	Preclinical phase	[348,349,350]
**pNL-DTΔN-GFP-RRE-SA**	HIV Rev cis-acting responsive sequence	Attenuated DTA variant	Non-integrating lentivirus	HIV-1 infection	Preclinical phase	[351]
**pA3-6PED**	PAX3 DNA responsive sequences	DTA	Cationic liposomes	ARMS	Preclinical phase	[352]
**petbz.ES.DT-A, pA.E-Sel.DT-A**	E-selectin *	DTA	Naked DNA-electroporation	Activated endothelial cells (Angiogenesis)	Preclinical phase	[353]
**GH-loxP-DT + CMV-Cre / GH-Cre**	Growth hormone (GH) *	DTA	Adenovorus	Pituitary Tumor	Preclinical phase	[354]
**BV-CG/ITR-DTA**	GFAP * + CMV enhancer + ITR of AAV	DTA	Baculovirus	glioma	Preclinical phase	[355]
**G1CEAPEANa, G1CEADTANa**	CEA *	PEA, DTA	Cationic liposomes	Colorectal carcinoma	Preclinical phase	[356]
**pRad51-DTA**	Rad51 *	DTA	Various transfection methods	Variety of cancer cells	Preclinical phase	[357]
**pAF-DTA, pAF5.1DTA**	AFP *	DTA	Cationic liposomes	HCC	Preclinical phase	[358,359]
**pTHA45, pTHA17**	Immunoglobulin heavy/κ-light chain *	DTA	Naked DNA-electroporation	B-Lymphoid Cells	Preclinical phase	[360]
**pTyrIII/DT-A, pMIA III/DT-A**	MIA, tyrosinase, *	DTA	Cationic lipids	Melanoma	Preclinical phase	[361]
**retro-1.3MBPppe, retro-1.3MBPpri**	MBP *	PE/RTA	Retrovirus	Glioblastoma	Preclinical phase	[362]
**pMSLN/DT-A**	MSLN *	DTA	Cationic polymer	Pancreatic cancer	Preclinical phase	[363]
**V3**	Hsp70B' * + HSEs	DTA, attenuated DTA variants	Cationic liposomes	Pancreatic cancer	Preclinical phase	[364]
**pLTR-DT**	p34 responsive sequences (BLV-LTR)	DTA	Cationic liposomes	BVL infected cells	Preclinical phase (veterinary use)	[365,366]

Abbreviations: DTA/DT-A: the catalytic A fragment of diphtheria toxin; PSA: prostate-specific antigen; hCG: human chorionic gonadotropin; ARMS: alveolar rhabdomyosarcoma; GFAP: glial fibrillary acidic protein; ITR: inverted terminal repeats, AAV: adeno-associated virus; PEA: truncated form of *pseudomonas* exotoxin A (domains II+III); CEA: human carcinoembryonic antigen; HCC: Hepatocellular carcinoma; hTER: human telomerase RNA, hTERT: human telomerase reverse transcriptase; AFP: human alpha-fetoprotein; MIA: Melanoma inhibitory activity; MBP: myelin basic protein; MSLN: Mesothelin; HSP: heat shock protein; HSEs: heat shock elements; hCG: human chorionic gonadotropin; CMV: cytomegalovirus; HIV: human immunodeficiency virus; LTR: long terminal repeat; BLV: Bovine leukemia virus. * Refers to the name of the gene/gene product whose transcriptional regulatory elements were used to drive the expression of a toxic gene in target cells.

### 3.1. Targeting Prostate Cancer

Prostate cancer (PCA) is the most common cancer diagnosed in men and a leading cause of cancer deaths [367]. Normal and malignant prostate epithelia specifically express the kallikrein protease prostate-specific antigen (PSA), a serine protease with trypsin- and chymotrypsin-like activities that is responsible for liquifaction of semen [368,369]. PSA gene regulatory regions are prime candidates to direct prostate-specific expression [370,371,372,373]. When placed under the transcriptional regulation of the PSA promoter, the diphtheria toxin A chain (DT-A) encoding gene which was delivered by an adenoviral vector showed strong inhibition of tumor growth in a PSA-producing prostate tumor xenograft mouse model while not affecting non-PSA producing tumor xenografts [339]. A PSA promoter-based lentiviral vector has also been used as a mean for DT-A gene delivery, leading to specific eradication of prostate tumor xenografts in nude mice following a single intratumoral injection. Repetitive injections were shown to inhibit the growth of recurrent tumors [374]. 

In another study, a nanoparticulate system based on the cationic poly(b-amino ester)polymer, C32 [375], was used as a DNA delivery system of a suicide gene encoding for a diphtheria toxin A chain (DT-A) under the transcriptional regulation of a prostate-specific modified human PSA promoter, PSE-BC [371]. The genetic material/cationic polymer complex was locally delivered into normal prostate and prostate tumors in mice, causing massive apoptotic prostate cell death, without damaging surrounding tissue [341]. 

In order to gain tighter control on the expression of the very potent DT-A toxin, Peng *et al* [340] utilized a dual expression controlling system that relies both on transcriptional regulation and DNA recombination [376,377,378]. On the basis of their system, the chimeric modified enhancer/promoter sequence of the human prostate-specific antigen (PSA) gene, PSE-BC [371], was used to regulate the expression of the *Saccharomyces cerevisiae* 2µ plasmid derived site-directed FLP recombinase. When prostate specific expression of FLP recombinase occurs, site directed DNA recombination that leads to DT-A gene expression takes place. The investigators showed eradication of PSA-expressing normal prostate cells and prostate cancer cells in culture, in xenografts and in a transgenic mouse model following adenoviral delivery of DNA encoding the prostate specific promoter-driven Flp recombinase and the Flp-responsive DT-A gene. Furthermore, Flp recombinase expression was shown to be regulated in a manner that correlates with the amount of PSA expression in these cells [340].

### 3.2. Targeting Ovarian Cancer

Ovarian cancer is a common malignancy in women, and causes more deaths than any other type of female reproductive tract cancer [379]. Lidor *et al* [342] have demonstrated specific protein synthesis inhibition in malignant ovarian cell lines transfected with plasmids encoding transcriptionally regulated diphtheria DT-A. The toxic gene transcription was regulated by elements of the human chorionic gonadotropin (hCG) promoter, a heterodimeric glycoprotein placental hormone involved in different pregnancy-promoting processes. Although principally produced by trophoblasts, hCG is also expressed in some malignant tumors of the ovary, uterus, testis, colon, liver, pancreas lung, and stomach [380,381,382]. 

Targeted expression of DT-A in ovarian cancer cells *in vitro* and in tumor cells in mouse models was performed by Huang *et al* [343] using poly(h-amino ester) polymers as a vector for nanoparticulate delivery of DNA. In these ovarian-specific antitumor constructs, the promoters of two genes, HE4 (WFDC2) and MSLN (which transcriptional activity is significantly enhanced in ovarian cancer cells relative to normal ovarian cells and cells in other tissues [383,384,385,386,387,388,389]) were chosen to target the expression of DT-A gene to ovarian tumor cells. Significant reduction in tumor mass and a prolonged life span of xenografts bearing mice were observed as a result of DT-A nanoparticles administration directly to subcutaneous xenograft tumors and to the peritoneal cavity. Moreover, treatment with DT-A nanoparticles resulted in more efficient suppression of tumor growth compared to clinically relevant doses of the standard chemotherapeutics cisplatin and paclitaxel, with minimal nonspecific tissue and blood chemistry toxicity [343]. 

Recently, Mizrahi *et al* [345] reported the use of transcriptional regulatory sequences of the H19 gene to drive the expression of DT-A specifically in ovarian tumor cells. H19 is a paternally imprinted, maternally expressed, oncofetal gene that encodes an RNA acting as "riboregulator" without a protein product. It is expressed at substantial levels in several different human tumor types, including epithelial ovarian cancer [390], but is only marginally or not at all expressed in normal adult tissues [391,392,393,394,395,396,397,398,399]. Cationic polymer PEI based delivery of DTA-H19 plasmid, encoding the toxic DT‑A transcriptionally controlled by regulatory sequences of the H19 gene, showed high killing potential in ovarian cancer cell lines and a significant tumor growth inhibition in animals [345]. In a later case study, DTA-H19 plasmid that was intraperitoneally injected into the peritoneum of a woman with advanced and recurrent ovarian carcinoma has been reported to yield a complete resolution of ascites following several infusions, with minimum adverse events [346]. Phase I/II study of DTA-H19 administered intraperitoneally in subjects with advanced stage ovarian cancer with evidence of symptomatic ascites is currently ongoing.

### 3.3. Targeting Bladder Cancer

Bladder cancer is the second most common urologic malignancy after prostate cancer. It is estimated that bladder cancer will account for 70,530 new cases of cancer and 14,680 cancer-related deaths in the United States during 2010 [400,401]. One of the most crucial enzymes in cell immortality and cancer is telomerase which maintains telomere length stability in almost all cancer cells [402]. Essential conserved core components of the human telomerase include the reverse transcriptase protein family member hTERT and the telomerase RNA hTER [403]. Following *in situ* hybridization analysis that showed high levels of hTER and hTERT expression in bladder tumors (while no signal was detected in normal tissue), transcription regulatory elements of these two genes were used for targeted gene therapy purpose by driving the expression of the toxic DT-A gene in bladder cancer cells. Experimental data demonstrated that transfection of bladder and hepatocarcinoma cell lines with DT-A expression plasmids under the control of hTER or hTERT regulatory elements resulted in cytotoxicity in accordance with the relative activity of these promoter elements in these cells [347].

In another study, expression of DT-A in subcutaneous injected syngeneic bladder tumor cell lines in mice was driven by the previously described H19 gene regulatory sequence (which is expressed in tumors derived from tissues (such as bladder) that exhibited the gene during embryonic development [404]). Intratumoral injection of the DNA vector as a calcium phosphate precipitate caused a significant suppression of subcutaneous tumor growth, with no obvious toxicity toward the host [405]. Significant suppression of tumor growth in animals and nearly complete ablation of the tumor in two human patients was also reported by the same group following intravesicle (into the bladder) administration of the DTA-H19 vector (which also showed high killing potential in ovarian cancer cells, see above) complexed with the transfection enhancer reagent Jet-PEI. No apparent toxicity toward the host was observed [406]. Phase I/II clinical studies in 18 patients with H19 over expressing superficial bladder cancer showed 22% complete response and 44% complete marker tumor ablation or a 50% reduction of the marker lesion after six treatments in which the DTA-H19 (BC–819) vector was administrated intravesically as a complex with polyethyleneimine. No dose limiting toxicity was observed and the most frequent adverse events were mild to moderate bladder discomfort, dysuria, micturition urgency, urinary tract infection, diarrhea, hypertension and asthenia [344].

### 3.4. Targeting Viral Infected Cells

HIV-1, the main cause of acquired immune deficiency syndrome (AIDS), has been the most studied infectious agent in the last 30 years. The HIV retroviral genome carries six regulatory genes, including Tat, Rev, Vpr, Vif, Vpu, and Nef. Of these genes, Tat encodes a protein that plays key roles in controlling productive and processive viral gene transcription. The Tat protein binds to the specific sequences of TAR (Transactivation Response Element) located in the 5’ LTR, one of two terminal repeated segments of the viral genome, and exerts its effect by increasing the rate of transcription of the nascent HIV RNA. The viral Rev protein was found to be required for expression of the viral late gene products. By binding to a secondary RNA structure, the Rev-responsive element (RRE), the Rev protein tethers partially spliced and unspliced viral RNAs encoding the late viral proteins to the cellular CRM-1-mediated nuclear-export pathway, leading to enhanced cytoplasmic levels of these RNAs and increased expression of the encoded proteins [407,408,409,410,411,412]. Applying the knowledge about these viral molecular mechanisms for regulated gene expression, Harrison *et al.* have demonstrated the use of a combination of Tat and Rev *cis*-acting responsive sequences for achieving enhanced expression of transgenes in cells expressing both regulatory *trans*-acting Tat and Rev proteins; while maintaining low basal expression in naive cells [413]. Substantially impaired HIV production, following HIV proviral DNA transfection of HeLa cells containing integrated HIV-regulated (Tat-Rev responsive) DT-A gene, was shown in a subsequent study [414]. A T-Cell line (H9) that was transduced with a recombinant retroviral vector encoding HIV regulated wild-type or attenuated DT-A gene also showed substantially long-term impaired ability to produce HIV virions upon transfection with proviral DNA or infection with laboratory or clinical HIV strains [348,415]. Using the same construct, significant protection against HIV infection (dependent both on the stock of HIV-1 used and on the dose) was also observed in the U937 cell line which exhibits many of the characteristics of tissue monocytes that serve as an important reservoir for the virus *in vivo* [349,416]. It was later reported that co-transfection of the HIV regulated DT-A construct (HIV-DT-A) with an HIV proviral DNA using cationic liposome-mediated gene delivery (“lipofection”) could prevent virus production in HeLa cells. However, although HIV-regulated genes were found to be expressed when transfected into chronically HIV infected cells, transfection with HIV-DT-A did not significantly reduce virus production in an already chronically or de novo HIV-infected cell population, probably due to the low percentage (~5%) of “lipofected” cells [350].

## 4. Protease Activated Toxins

### 4.1. Extracellular Protease Activated Toxins

Cytotoxic activity of PE, DT and other bacterial toxins (like the anthrax toxin that will be described in the following section) depends on a proteolytic cleavage taking place in an early step of the intoxication process [122,123,157,158,417,418,419,420]. Since different disease-related cells are sometimes associated with a distinguished extracellular proteolytic activity, as will be discussed shortly, it is conceivable that replacing the natural cleavage site of a toxin with that of a disease-related protease may confer the new molecule with the ability to specifically eradicate disease-related cells (Figure 1). Several studies in which such molecules have been developed and preclinically evaluated, classified by their targets, are described in the following chapter. Information about the reviewed and other extracellular activated toxins is summarized in Table 3.

**Table 3:** Preclinically Evaluated/under Evaluation Protease Activated Toxins.

Construct Name	Activating Protease	Protease Localization	Components	Toxin Source	Activation Mode	Target	References
**PA-L1/L2 + FP59**	MMPs (mainly MMP2 and MMP9)	Extracellular	PA (modified) +FP59	Anthrax + PE	Binding and translocation of the toxic moiety	MMPs expressing tumor cells	[421]
**PA-L1 + LF**	MMPs (mainly MMP2 and MMP9)	Extracellular	PA (modified) +LF	Anthrax	Binding and translocation of the toxic moiety	Tumor vasculature; MMPs expressing tumor cells with V600E B-Raf mutation	[422,423,424]
**PrAg-U2 + FP59**	uPA	Extracellular	PA (modified) +FP59	Anthrax + PE	Binding and translocation of the toxic moiety	Tumor cells with receptor-associated uPA activity	[425,426,427,428,429]
**PrAg-L1-I210A + PrAg-U2 R200A + FP59**	MMPs + uPA (both required)	Extracellular	PA (modified) +FP59	Anthrax + PE	Binding and translocation of the toxic moiety	MMPs expressing tumor cells with receptor-associated uPA activity	[430]
**UFT3**	PSA	Extracellular and intracellular	Ubiquitin (mutant), saporin	Saporin	Toxin stabilization	Prostate cancer cells	[431]
**DTU2GMCSF**	uPA	Extracellular	DT_388_ (modified), GM-CSF	DT	Translocation of the toxic moiety	AML cells (the toxin is targeted also by fusion to GM-CSF)	[432]
**FLD/MM, FLD/YV**	HIV-1 protease	Intracellular	PA + LF_N_-DTA	Anthrax + DT	Toxin stabilization	HIV-1 infected cells	[433]
**TAT-Pro-HIV-p2/NC, TAT-Pro-HIV-MA/CA**	HIV-1 protease	Intracellular	HIV-1 TAT transduction peptide, Maize RIP (modified)	Maize RIP	Enhancement in the enzymatic activity of the toxic moiety	HIV-1 infected cells	[434]

Abbreviations: PE: *pseudomonas* exotoxin A; DT: diphtheria toxin; DTA/DT-A: the catalytic A fragment of diphtheria toxin; DT_388_: truncated form of DT; MMPs: matrix metalloproteinases; RIP: ribosome inactivating protein; PA/PrAg: anthrax protective antigen; FP59: anthrax toxin lethal factor residues 1–254 fused to the ADP-ribosylation domain of PE; PA: anthrax toxin protective antigen; LF: anthrax toxin lethal factor; uPA: urokinase plasminogen activator; NSCLC: non–small cell lung cancer; PSA: prostate-specific antigen; HIV: human immunodeficiency virus; AML: acute myeloid leukemia; GM-CSF: granulocyte macrophage colony-stimulating factor, LF_N_: amino acids 1-255 of anthrax toxin lethal factor.

#### 4.1.1. Targeting Matrix Metalloproteinases (MMPs) Overexpressing Tumor Cells

Matrix metalloproteases (MMPs) are a multigene family of zinc-dependent endopeptidases, which are secreted as latent pro-enzymes and have the capacity to degrade components of the extracellular matrix (ECM) following activating cleavage. In addition, MMPs have the ability to process molecules such as growth factors, receptors, adhesion molecules, other proteinases and proteinase inhibitors. MMPs are basically divided into distinct subclasses according to their substrate specificity and cellular localization: the secreted soluble collagenases, gelatinases, stromelysins and matrilysins; and the membrane-type MMPs which are integral plasma membrane proteins capable of activating MMPs. For certain family members, including some of the membrane-associated MMPs, activating cleavage can be achieved intracellularly by the protease furin. For other MMPs, however, activation is executed by extracellular proteases such as plasmin or other MMPs. While restricted to only a small number of normal cells, several MMPs are overexpressed by different kinds of solid tumors and have been implicated in the ECM degradation associated with tumor growth, angiogenesis and invasiveness [435,436,437,438,439]. 

**Figure 6:**
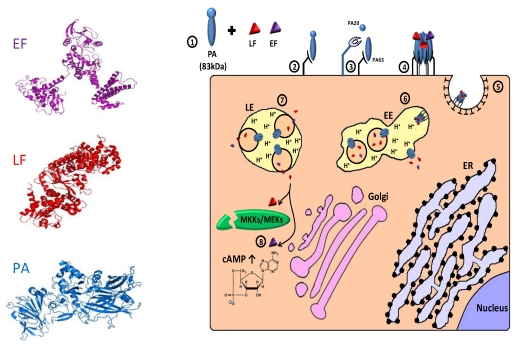
Cellular trafficking of anthrax toxin. 1. The toxins are secreted as 3 polypeptides: protective antigen (PA; 83 kDa), lethal factor (LF; 90 kDa) and edema factor (EF; 89 kDa) (see 3D structure (PDB Entry: PA- 1acc; LF-1j7n; EF- 1y0v). In the left panel, the colors of the different proteins correspond to those in the scheme; 2. PA binds to cellular receptor (ATR/TEM8; CMG2); 3. Cell-surface furin protease cleaves PA into an N terminal PA63 (63 kDa) and C terminal PA20 (20 kDa) fragments; 4. Receptor bound PA63 self associates into a homoheptamer (“prepore”) that can bind up to 3 molecules of LF and/or EF; 5. The complex internalized via clathrin-dependent receptor mediated endocytosis; 6. In the early endosomes (EE), the complex is sorted to the vesicular region and preferentially incorporated into intraluminal vesicles. The acidic environment of the endosome induces a conformational change in the prepore that turns into a channel/pore and functions in the translocation of LF and/or EF to the lumen of intraluminal vesicles or to the cytoplasm; 7. Following transportation to late endosome (LE), back fusion of intraluminal vesicles with the limiting membrane delivers the “trapped” toxic factors to the cytoplasm; 8. In the cytoplasm, LF functions as a zinc metalloproteinase that cleaves the N termini of MKK/MEK proteins, blocking their signaling activity. EF acts as a Ca^2+^/calmodulin activated adenylate cyclase that dramatically elevates cytoplasmic cAMP level and consequently disrupts normal cellular activities.

Anthrax toxin (AnTx) is a major virulence factor secreted by the gram-positive, spore-forming bacterium *Bacillus anthrachis.* The toxin, which damages cells and impairs host defenses, belongs to a family of toxins called “binary toxins” which are characterized by consisting of minimally two discrete nontoxic proteins that must be combined to elicit toxicity. AnTx consist of three non-toxic plasmid‑encoded multidomain proteins: protective antigen (PA/PrAg; 83 kDa), lethal factor (LF; 90 kDa) and edema factor (EF; 89 kDa) (for review, see [440,441,442]). Intoxication begins with the binding of PA to either of its two known cellular receptors ATR/TEM8 [443] and CMG2 [444]. PA then undergoes an activating cleavage by a member of the furin family of cellular proteases [418,420], into an N terminal PA63 (63 kDa) and C terminal PA20 (20 kDa) fragments. Following cleavage, receptor-bound PA63 self associate to form a ring-shaped homoheptamer [445], called the prepore, which may then form complexes with up to three molecules of LF and/or EF (each bound LF/EF molecule “occupies” two neighboring PA subunits). The resulting complexes, which are known as lethal toxin (LeTx) and edema toxin (EdTx), respectively, are then internalized via clathrin-dependent receptor-mediated endocytosis [446,447] and delivered to early endosomes where the complex is sorted to the vesicular regions and preferentially incorporated into intraluminal vesicles [448]. Subsequently, the prepore undergoes an acidic pH-dependent conformational change to form a cation-selective, ion-conducting channel [449,450,451]. This channel/pore is thought to participate in the unfolding of LF and EF, and functions in their translocation into the lumen of the intraluminal vesicles or into the cytoplasm, probably with the aid of cytosolic components [452,453,454,455,456]. Following transportation to late endosomes, back fusion of intraluminal vesicles with the limiting membrane delivers the toxic factors, which were “trapped” inside the vesicles lumen, to the cytoplasm [448] (for review about the cellular routing of the anthrax toxin, see [442,457]). Once in the cytoplasm, LF functions as a zinc metalloproteinase that specifically cleaves the N-termini of MKK/MEK proteins (kinases of mitogen-activated protein kinases), blocking their signaling activity [458,459,460,461,462,463,464,465,466]. EF is a Ca^2+^/calmodulin activated adenylate cyclase (AC) that acts by elevating the intracellular level of cyclic AMP (cAMP), upsetting water homeostasis, destroying the delicate balance of intracellular signaling and impairing neutrophil functions [440,467,468,469,470,471,472] (Figure 6). 

Liu *et al* [421] were the first to introduce and apply a new concept in which replacing the natural furin cleavage site in anthrax PA protein (such cleavage must occur on the cell surface in order to achieve binding and internalization of a catalytic LF and EF) with a sequence recognized by a tumor‑associated protease may confer the recombinant molecule with the ability of targeting the protease overexpressing cells. To this end, in two mutated PA proteins, PA-L1(named also PrAg-L1) and PA-L2, the natural furin recognition site was replaced by sequences susceptible to cleavage by MMP-2 (gelatinase A) and MMP-9 (gelatinase B) that are reported to be related to invasion and metastasis in various human cancers [473,474,475,476]. The toxic catalytic polypeptide that has been used in this study was a fusion between the ADP-ribosylation domain of *Pseudomonas* exotoxin A and amino acids 1–254 of LF (LF_N_), which contains the PA binding domain that proved sufficient to achieve translocation of a fused “passenger” polypeptide to the cytosol of cells in a PA-dependent process [477,478,479]. By combining the re-engineered PA with the fusion toxic polypeptide (which was denoted FP59), the researchers have demonstrated selective killing of MMP-overexpressing human tumor cell lines while sparing nontumorigenic normal cells. Protection against challenge from PA‑L1/L2 plus FP59 by MMP inhibitors further demonstrated that cell killing is highly dependent on the MMPs activity expressed by the tumor cells. Furthermore, specific eradication of MMP overexpressing tumor cells in a co-culture model indicated that PA activation occurred on the tumor cell surface and not in the culture supernatant. Activation of MMPs on the cell membrane by plasmin and/or membrane-anchored MMP, together with binding to cell surface receptors, were proposed as factors that may contribute to the retention of soluble active MMPs on the surface of tumor cells [421].

In contrast to the enzymatically active polypeptide of PE, DT and RIPs, which strongly inhibit protein synthesis causing the death of the intoxicated cell (see above), inactivation of mitogen-activated protein kinase kinases by the action of anthrax lethal factor (LF) was found to selectively kill cells in which an activated MAPK pathway is required for their survival. This is similar to observed for cells bearing the V600E mutation in B-Raf, a serine/threonine kinase immediately upstream of MEK1/2 in the of the ERK MAPK cascade. The mutation, which was demonstrated mainly in melanoma but also in other human malignancies, “locks” the molecule in a constitutively active state, making the cell dependent on the constitutive activation of the ERK pathway for survival [480,481,482,483]. However, although specific toxicity toward B-Raf mutant melanoma cells has been observed both *in vitro* and in xenograft melanoma tumors in mice [481,482,483,484], development of LeTx variants with lower *in vivo* toxicity and high tumor specificity would be required for use in human cancer patients [485].

For the purpose of using LF as a targeted toxic polypeptide with superior specificity, a modified LeTx (PA-L1/LF) composed of LF and MMP activated PA protein was tested in mice xenograft models of human tumors. As expected, the PA-L1/LF showed lower toxicity to mice than wild-type toxin and has a potent anti-tumor activity. Surprisingly, anti-tumor activity was observed not only against human melanomas with B-Raf V600E mutation (due to direct toxicity against these cells), but also against other human tumor types including colon and lung carcinomas, and mouse tumors, regardless of their B-Raf status. Tumor histology and *in vivo* angiogenesis assays suggested that these effects can be attributed to indirect targeting of tumor vasculature and angiogenic processes (expression of MMPs has been shown to be upregulated in angiogenic lesions [486,487,488,489]), in which mitogen-activated protein kinase (MAPK) signaling pathways play a central role [422,490]. Later *in vitro* studies on endothelial proliferation, invasion, and tube formation showed that activated LeTx acts by impairing microvascular endothelial cell invasion and migration in the absence of endothelial cell death [423]. Another intriguing observation was that the PA-L1/LF administration showed higher anti-tumor efficacy than does the wild-type toxin, probably because of greater bioavailability and longer half-life of PA-L1(relative to PA) in circulation [422]. 

Treatment with PA-L1/LF also delayed tumor growth and improved long-term survival in mice with orthotopically implanted anaplastic thyroid carcinoma (ATC) xenografts. The antitumorogenic effect was comparable with that achieved by the multikinase inhibitor, sorafenib, which is currently under evaluation for clinical use in patients with papillary thyroid carcinoma (PTC). As was already found in previous studies [422,423], the therapeutic effect of PA-L1/LF was mediated by the potent antiangiogenic activity of the drug, which did not cause any significant systemic toxicity [424].

#### 4.1.2. Targeting Malignant Cells Overexpressing the Urokinase Plasminogen Activator System

Plasminogen is an inactive precursor of plasmin, a serine protease present in plasma and extracellular fluid and is active in the process of fibrinolysis and degradation of extracellular matrix (ECM) components [491]. One of the naturally occurring plasminogen activators is the urokinase-type plasminogen activator (uPA), a serine protease secreted as an inactive pro-enzyme (pro-uPA) that efficiently converted by plasmin or kallikrein into uPA, which in turn cleaves plasminogen to generate the active plasmin in a positive feed-back mechanism. The catalytic efficiency of these reactions is further increased upon binding of pro-uPA/uPA and plasminogen/plasmin to membranal receptors, resulting in reciprocal zymogen activation on the cell surface. The initial proteolytic event leading to the initiation of plasmin generation on the cell surface is still under study [492,493,494,495,496,497,498]. While quite restricted in normal tissues, expression of pro-uPA and its receptor, uPAR, was found to be elevated in a significant number of solid tumors. Moreover, the presence of this proteolytic system which confers the tumor mass with the ability to degrade ECM proteins is associated with increased tumor tissue invasiveness and metastatic potential [499,500]. 

To target tumor cells that overexpress the urokinase plasminogen activator system, the furin cleavage site in anthrax PA protein was replaced by sequences that are specifically cleaved by uPA. The modified PA was then combined with FP59, a fusion between the ADP-ribosylation domain of *Pseudomonas* exotoxin A and amino acids 1-254 of LF (see above). In the presence of pro-uPA and plasminogen, the recombinant complex (PrAg-U2 + FP59) was demonstrated to be activated selectively on the surface of uPAR-expressing tumor cells from a broad range of human cancers of different origins, and ultimately resulted in specific tumor cell eradication [425,427]. Subsequent *in vivo* studies have demonstrated efficient suppression of different murine tumors growth and eradication of established tumors with limited toxicity to normal tissue upon local and systemic administration of the complex [426,428]. 100% tumor regression and 30% complete histologic remission after systemic administration of tolerated doses of PrAg-U2+FP59 were documented in an *in vivo* athymic nude mouse model of human non-small cell lung cancer (NSCLC) [429]. 

The requirement of two activating proteolytic events by two different tumor-associated proteases may confer the toxic agent with superior cell-type specificity and further attenuate its toxicity to normal tissues. In an elegant work performed by Liu *et al* [430], an intermolecular complementation approach was used for the targeting of tumor cells having both MMP and uPA activities which are overproduced by tumor tissues and are implicated in cancer cell growth and metastasis [435,436,437,438,439,499,500]. To this end, the authors exploited the findings that the anthrax LF binding site spans two adjacent monomers of cleaved PA in the oligomeric prepore. Each monomer contains three subsites which play a role in the binding of LF. However, the functional LF binding site is composed of a combination of subsites contributed by two adjacent subunits: subsite I and III form one monomer and subsite II from the neighboring subunit [501,502]. By mutating LF binding subsite III on an MMP activated PA (PrAg-L1) and subsite II on uPA activated PA (PrAg-U2), assembly of PA heptamer in which every LF binding site contains the inactivating subsite III or subsite II mutation will occur following activating cleavage by either MMP or uPA, respectively. However, adding a mixture of these modified PA proteins to cells that have both MMP and uPA activities would generate two subunits that can randomly assemble into a heptamer in which up to three functional LF binding sites may be generated by intermolecular complementation between the two types of mutated subunits. In order to test the hypothesis that requirement of two tumor-associated activating proteolytic events may attenuate toxicity to normal tissues, an *in vivo* toxicity assay in mice was performed by intraperitoneal administration of a mixture containing subsite III mutated PrAg-L1 (PrAg-L1-I210A), subsite II mutated PrAg-U2 (PrAg-U2-R200A) and the toxic FP59 chimeric polypeptide. Indeed, results showed decreased toxicity in comparison to administration of a mixture of PrAg-L1, PrAg-U2 and FP59 (where either MMP or uPA proteolytic activity was sufficient to generate a functional LF binding prepore).

When the combination of PrAg-U2-R200A and PrAg-L1-I210A (with FP59) was evaluated in treatment of three mouse tumor types (B16-BL6 melanoma, T241 fibrosarcoma, and LL3 Lewis lung carcinoma), a strong anti-tumor activity was observed. In contrast, the tumors showed little or no response to treatment with the individual proteins, demonstrating the necessity of intermolecular complementation between these engineered PA/PrAg proteins for the execution of a potent tumoricidal activity. In an anti-tumor efficacy comparing assay, the combination of PrAg-U2-R200A and PrAg‑L1‑I210A was at least as effective as an equivalent dose of PrAg-U2. However, given that the maximum tolerated dose of PrAg-U2-R200A/PrAg-L1-I210A is higher than that of PrAg-U2, the complementing mixture achieves higher tumor specificity [430].

### 4.2. Intracellular Protease Activated Toxins

The life cycle of many viruses depends upon viral proteases for the cleavage of high molecular weight precursor viral proteins in order to yield functional products or by catalyzing the processing of the structural and non-structural proteins that are necessary for assembly and morphogenesis of viral particles. A partial list of human disease-associated viruses encoding protease(s) in their genomes include flaviviruses such as: hepatitis C virus (HCV), West Nile virus (WNV), dengue fever virus (DFV) and yellow fever virus (YFV); retroviruses such as HIV-1; picornaviruses such as coxsackievirus, poliovirus and hepatitis A virus; nidoviruses such as coronaviruses (CoV), including the severe acute respiratory syndrome (SARS) causative SARS-CoV; and herpesviruses such as varicella-zoster virus (VZV) and Epstein-Bar virus (EBV) [503,504,505]. As expression of the viral protease distinguishes an infected cell from surrounding healthy tissue, limiting virus production and spread by a viral-protease activated toxin that specifically eradicates infected cells may comprise an attractive antiviral approach (Figure 1). Selected preclinical studies in the field are described in the following chapter. Information about the reviewed intracellular activated toxins is also summarized in Table 3.

#### 4.2.1. Targeting HIV Infected Cells

The half-lives of proteins in a living cell range from a few seconds to many days. Features of proteins that confer metabolic instability are called degradation signals, or degrons. The N-end rule degron (N-degron), which was the first to be discovered, relates the *in vivo* half-life of a protein to the identity of its amino-terminal residue. In eukaryotes, the N-degron comprises at least two determinants: a destabilizing N-terminal residue and internal lysine/s that function as acceptor site/s for the formation of a multi-ubiquitin chain that “marks” the ubiquitin-protein conjugates as a substrate for proteosomal degradation. Destabilizing residues in mammalian cells can be hierarchically divided into three classes: Primary (type 1 (Arg, Lys or His) or type 2 (Ile, Leu, Phe, Tyr or Trp)), secondary (Asp, Glu or oxidized Cys) and tertiary (Asn, Gln or Cys) (reviewed in [506,507,508,509,510,511]). 

In a comprehensive study by Falnes *et al* [512], the *in vivo* stability and cytotoxicity of diphtheria toxin-based polypeptides, artificially modified to initiate with short sequences derived from the FLAG peptide epitope differing only in their N-terminal amino acids, were assessed. According to their findings, when the first N terminal amino acid of the modified toxins were Phe, Tyr, Trp, Asp, Asn, Glu, Gln, Lys, Arg or His, the proteins were highly unstable, with half-lives in the range 0.2–1.6 hours, while the toxins initiated with either of the remaining amino acids were considerably more stable, with half-lives ranging from 3 to >12 hours.

An *in vitro* study in reticulocyte lysates indicated that the ten N terminal amino acids that confer the toxin with short *in vitro* half life were also the most destabilizing ones *in vivo*. Moreover, cytotoxicity assays in Vero cells showed a clear correlation between the *in vivo* stability and the cytotoxicity of the proteins, although the difference in cytotoxicity between the wild-type DT-A fragment and the least stable mutant was only ~20-fold in compared to ~100-fold difference in intracellular half-life [512].

Alexander Varshavsky suggested previously the construction of a new kind of toxins where a signal that inactivates the toxin, e.g. a degradation signal, can be cleaved off by a viral protease, resulting in selective intoxication of virally infected cells. He denoted such toxins “sitoxins” (signal-regulated, cleavage mediated toxins) [506]. For the construction of such viral-protease activated sitoxins, Falnes *et al* designed fusion proteins composed of a FLAG peptide containing an N-terminal phenylalanine (a destabilizing amino acid according to the N-end rule), followed by an HIV-1 protease (HIV-1 PR) cleavage sites that is positioned upstream to a chimeric sequence between DT-A and the first PA-binding 255 amino acids of the anthrax LF, the LF_N_ fragment, which was also shown to be destabilized by the addition of a degradation signal for N-end-rule-mediated degradation [513]. The rationale behind this construct was that following anthrax PA-mediated translocation into the cytoplasm of uninfected cells, the modified toxin would be rapidly degraded by virtue of its destabilizing N-degron, resulting in attenuated cytotoxic activity against these uninfected cells. In contrast, cleavage of the construct by the HIV-1 protease whose activity has been documented in the cytosol of acutely infected cells [514,515,516,517,518] would result in the removal of the destabilizing N degron from the chimeric toxin, exposing a new N terminal amino acid. As HIV-PR cleavage in each of its two recognition sequences that were chosen (TATIM*MQRG or VSQNY*VIVQ) generates a stabilizing N terminal residue (methionine or valine, respectively) according to the N-end rule, a long-life toxin with a potent cytotoxic activity is generated in HIV infected cells which leads to their destruction. Indeed, *in vitro* and *in vivo* simulating experiments showed that when the constructs were pre-digested with HIV-PR, both stability and cytotoxicity of the chimeric toxins was considerably augmented, proving that the concept of stabilizing a protein through specific proteolytic removal of a degradation signal works in practice. However, no selective eradication of HIV infected cells was observed following treatment with non pre-treated constructs, probably because of low cytosolic HIV-1 PR activity (no proteolytic processing of the constructs were detected in HIV-infected cells) [433]. 

In another study, which was performed by Law *et al* [434], HIV protease-activated toxins were designed on the basis of the type III maize ribosome-inactivating protein, which in its native form is synthesized as inactive precursor (pro-RIP) and is activated by proteolytic removal of a 25 amino-acid inhibitory internal peptide *in-planta* [218,219]. In this work, the first and the last 10 residues of the internal inactivation region were replaced with two HIV-PR recognition sequences, and an 11 amino‑acids transduction peptide derived from the HIV-1 Tat protein was fused to the N-termini of the modified RIPs for promoting cell entry [519]. These modifications resulted in the generation of re‑engineered pro-RIPs which underwent an efficient cleavage *in vitro* by recombinant HIV-PR or *in vivo* (in HIV infected cells) by the viral encoded protease. Upon treatment of infected cells, the *N*‑glycosidase and anti-viral activities of the modified cleavable RIPs were found to be higher than those of an uncleavable-nonactivated pro-RIP and resembled those of an activated mutant in which the inhibitory region was genetically removed. The cytotoxic activity of the cleavable RIPs against infected cells was not reported, but cytotoxicity toward uninfected cells was found to be lower than that of the activated mutant. Thus, the fact that the cleavable toxins behave more like a constitutively active form toward infected cells and in a similar way to a non-activated form toward uninfected cells, may suggest that they are specifically activated by the HIV protease and demonstrates the possibility of using this cleavable internal inactivation region as a switch to activate the toxin inside infected cells [434].

## 5. Concluding Remarks

Treating cancer and chronic viral infections are among the most challenging goals in modern medicine. The recent integration of modified plant and bacterial toxins based therapies (and particularly immunotoxins with anti-tumor activity) into clinical research and application adds new weapons to the arsenal against these refractory diseases. Cumulative knowledge about toxins’ structure and mechanism of action, as well as recent progress and breakthroughs in the fields of cell biology, immunology, virology, molecular biology and nanotechnology, enabled the development of different targeting strategies that are crucial for converting a lethal toxin into a therapeutic agent (see Figure 1). However, clinical application of engineered toxins still faces many challenges. Two major problems associated with systemic administration of immunotoxins are: 1. lack of specificity resulting from the presence of the target antigen/receptor also being present on healthy tissue (“target dependent toxicity”); 2. Undesired intoxication of healthy tissue due to the immunotoxin binding to cell surface components rather than specifically to its target antigen/receptor (“target independent toxicity”). Whereas the first problem directly relates to the target and its expression/body-distribution profile; the second relates to the nature of the therapeutic agent itself. 

The most common toxicities in patients treated with immunotoxins are vascular leak syndrome (VLS) and hepatotoxicity, caused by non-specific binding of the toxic or the targeting moiety of the immunotoxin to endothelial and hepatic cells, respectively (for review, see [11,520,521]). Regarding this issue, Baluna *et al* have found that sequences of three amino acids in ricin toxin (and in other proteins associated with vascular leak syndrome) might cause endothelial damage by a mechanism involving disruption of integrins, independently of the “classical” cell killing mediated by the toxin’s enzymatic activity [522]. By screening a panel of recombinant ricin A chains with mutations in this sequence or in amino acids flanking it in the three-dimensional structure, a mutant form of RTA showing diminished VLS in mice was isolated [523,524].

While pharmacokinetics issues and non-specific toxicity are common problems associated with the development of many drugs and particularly with the development of chemotherapeutic agents; immunogenicity is a major challenge distinguishing immunotoxins from small molecule-based therapy. Immunotoxins generally contain at least one non-human component which might be the bacterial/plant derived toxic moiety and/or an antibody from animal origin. Consequently, anti-drug antibody formation may be induced in immune-competent patients, resulting in a compromised treatment efficiency caused by the decrease in the level of circulating functional agent. The issue of human-anti mouse antibody (HAMA) formation against immunotoxins, in which the targeting moiety is a monoclonal antibody produced by hybridoma cells, has been overcome to a great extent by the development of chimeric, humanized and fully human antibodies using recombinant DNA technology (reviewed by [525,526]). Attempts to use polyethylene glycol (“PEGylation”) [527,528,529] and immunosuppressive agents [105,107,530,531,532] have led to different success rates in reducing the immunogenic response against non-human components of immunotoxins. Deletion of B-cell and T‑cell epitopes located on the surface of the toxic moieties is another option for reducing their immunogenicity. Recent studies conducted by Onda *et al* revealed seven major conformational B-cell epitopes on the PE38 molecule [533]. By mutation of specific large hydrophilic amino acids on the surface of the PE38 component in the immunotoxin BL22 (see above), most of the B cell epitopes were eliminated and a new fully functional immunotoxin which is significantly less immunogenic to mice was created [534]. Taking advantage of this knowledge, Oh *et al* have created a bispecific cytotoxic polypeptide in which human epidermal growth factor (EGF) and interleukin-4 (IL-4) are linked to the B-cell epitopes-deleted PE38. When administrated to a mouse model of metastatic breast carcinoma, immunogenicity was reduced by about 90% (in comparison to the non-mutated construct) with no apparent loss of anti-tumor activity [535].

Concerning protease activated toxins and toxin based suicide gene therapy, clinical data is scarce as most developments in this field have not been evaluated clinically. However, it is conceivable that the specificity issues will remain relevant also for these agents as the transcription or proteolytic activity that execute their toxicity are rarely associated exclusively with a diseased cell, but rather, as was previously mentioned, have also a physiological role in healthy tissue. As in the case of immunotoxins, immunogenicity may also be a problem that must be overcome when treating immuno-competent patients with protease activated toxins or toxin based suicide gene therapy, either because of the immunogenicity of the cleavable toxin or the immunogenicity of the viral vecto. 

In conclusion, toxin-based therapy is a versatile and dynamic research area with a great application potential, as demonstrated in this review. However, further research is required in order to improve the efficiency and safety of toxin-based agents. Investments in the development of delivery and targeting techniques are definitely required in order to achieve this goal, but nevertheless the basic research on the structure and mechanism of natural toxins should not be abandoned. The deeper our knowledge about this unique family of secreted polypeptides, the easier for us to harness their great potential for our own benefit.
